# The Role of Blood Platelets in Experimental Metastases

**DOI:** 10.1038/bjc.1973.168

**Published:** 1973-11

**Authors:** P. Hilgard

## Abstract

After the intravenous injection of Walker 256 tumour cells into rats the platelet count decreased rapidly and remained low during the following period of observation. The platelet decrease was closely related to the number of cells injected. Intra-arterial tumour cell injections required a considerably higher tumour cell count to produce a comparable thrombocytopenia. Non-viable tumour cells and tumour cell fragments induced a similar decrease of circulating platelets. Neither viable tumour cells nor tumour cell fragments aggregated rat platelets *in vitro.* The presence of fibrin monomers in tumour cell injected animals suggested intravascular fibrin deposition; the plasma fibrinogen level, however, did not decrease significantly. Isotope studies using ^51^Cr labelled platelets revealed a rapid disappearance of the platelets from the circulation and their trapping in the lung—the primary site of tumour cell lodgement. Dipyridamole and ancrod pretreatment did not influence the decrease of platelets and their accumulation in the lung after tumour cell injection. In contrast, heparin completely prevented the thrombocytopenia and the platelet trapping in the lung. From the present experiments it is concluded that embolic tumour cells lead to early endothelial damage, resulting in local thrombin formation with subsequent irreversible platelet aggregation.


*Br. J. Cancer* (1973) **28**, 429

### THE ROLE OF BLOOD PLATELETS IN EXPERIMENTAL METASTASES

P. HILGARD*

*From the Department of Haematology, Royal Postgraduate Medical School, London W12 0HS*

Received 11 May 1973. Accepted 21 July 1973

**Summary.—After the intravenous injection of Walker 256 tumour cells into rats the platelet count decreased rapidly and remained low during the following period of observation. The platelet decrease was closely related to the number of cells injected. Intra-arterial tumour cell injections required a considerably higher tumour cell count to produce a comparable thrombocytopenia. Non-viable tumour cells and tumour cell fragments induced a similar decrease of circulating platelets. Neither viable tumour cells nor tumour cell fragments aggregated rat platelets *in vitro*. The presence of fibrin monomers in tumour cell injected animals suggested intravascular fibrin deposition; the plasma fibrinogen level, however, did not decrease significantly. Isotope studies using $^{51}Cr$ labelled platelets revealed a rapid disappearance of the platelets from the circulation and their trapping in the lung—the primary site of tumour cell lodgement. Dipyridamole and ancrod pretreatment did not influence the decrease of platelets and their accumulation in the lung after tumour cell injection. In contrast, heparin completely prevented the thrombocytopenia and the platelet trapping in the lung. From the present experiments it is concluded that embolic tumour cells lead to early endothelial damage, resulting in local thrombin formation with subsequent irreversible platelet aggregation.**

EXPERIMENTAL evidence has accumulated indicating that the activation of the blood clotting mechanism at the site of tumour cell lodgement is a part of the pathology of metastatic tumour growth and *in vivo* observations in the rabbit ear chamber demonstrated clot formation around embolic tumour cells (Wood, Holyoke and Yardley, 1961). Inhibitors of blood coagulation or fibrinolytic agents were shown to reduce blood bone metastases in experimental conditions (Wood *et al.*, 1961; Cliffton and Agostino, 1965; Agostino and Cliffton, 1965; Ryan, Ketcham and Wexler, 1968). It has been concluded that fibrin formation around adherent tumour cells is an important step in the establishment of haematogenous metastases.

It was assumed that blood platelets are involved in tumour cell lodgement as well as participating in thrombus formation, and that they may represent an additional mediator of metastasis formation (Gasic, Gasic and Stewart, 1968). The present investigations were designed to obtain further information about the behaviour of blood platelets after administration of experimental tumour cells into the blood stream. The Walker carcinosarcoma 256 of the rat was chosen for these experiments because the ultrastructure of its intravascular behaviour is fairly well investigated (Griffith and Salsbury, 1963; Jones, Wallace and Fraser, 1971; Warren and Vales, 1972). The use of the ascitic form provides a suspension of single, viable tumour cells which is easily obtained. After the intravenous injection the majority of the cells are arrested in the lungs, representing the primary site of metastatic

* Present address: Universitätsklinikum (Tumorforschung), D-43 Essen 1, West Germany.

tumour growth (Cliffton *et al.*, 1971). Since the Walker carcinosarcoma 256 is an allogeneic tumour, immunological reactions which may complicate the experimental results have to be taken into account. However, only the initial phase of tumour cell lodgement was studied in the present experiments and for that purpose the Walker carcinosarcoma 256 was considered to be an acceptable tumour system.

### MATERIALS AND METHODS

Female Wistar derived rats (inbred since 1953 at the Royal Postgraduate Medical School, London), aged 5–6 weeks and weighing 110–150 g, were used throughout the experiments.

The Walker carcinosarcoma 256, originated from a spontaneously grown mammary adenocarcinoma (Stewart *et al.*, 1959), was transferred to the ascitic form (Agostino and Cliffton, 1968) using sterile techniques. Subsequently the tumour was kept in the ascitic form by intraperitoneal inoculation of $2 \times 10^6$ freshly obtained tumour cells into female rats (110–150 g). The intramuscular transplantation of $5 \times 10^6$ tumour cells resulted in the animals' death 9–10 days later, at which time the solid tumour had a diameter of 3–4 cm. Six days after the intraperitoneal inoculation the ascites was harvested for the experiments. After washing and differential centrifugation at 4°C with buffered saline (pH 7·4) containing 5% dextrose, the tumour cell suspension was adjusted to the desired cell count with buffered saline–5% dextrose.

The viability of the tumour cell suspensions, or the suspensions of cell fragments, was assessed by intramuscular transplantation of $5 \times 10^6$ cells into healthy control animals.

Tumour cell fragments were prepared by triple freezing and thawing of a tumour cell suspension with a known cell count. Finally, the fragments were washed and resuspended in buffered saline to the original volume. The supernatant was kept and frozen for later platelet aggregation studies.

All injections were made during methoxyflurane (Penthrane, Abbott Laboratories Ltd.) anaesthesia into the lateral tail veins of the animals. In some instances tumour cell suspensions were injected into the thoracic aorta *via* a polyethylene cannula inserted into the femoral artery.

Blood samples were obtained under methoxyflurane anaesthesia by aortic puncture and the blood was allowed to flow directly into a polyethylene test tube containing 2 mg of EDTA. Serial platelet counts were performed from tail vein blood.

Platelet counts were made in duplicate (Brecher and Cronkite, 1950) and the plasma fibrinogen was estimated according to the method of Ratnoff and Menzie (1951). The screening for fibrin monomers in plasma was performed with the protamine precipitation test (Sanfelippo, Stevens and Koenig, 1971).

Platelet aggregation was measured in a platelet aggregation meter (Evans Electroselenium Ltd.) using rat platelet rich plasma and 3·8% sodium citrate (1 : 10) as anticoagulant. Aggregating substances were suspensions of freshly prepared collagen particles, fresh tumour cell suspensions, tumour cell fragments and tumour cell extracts. After addition of the test sample the decrease of light transmission was recorded.

Rat platelets from healthy donor animals were labelled with $^{51}Cr$ as described by Dacie and Lewis (1968). 0·5 ml of platelet rich plasma, containing approximately $9 \times 10^5$ labelled platelets per $mm^3$ (corresponding to approximately $8 \times 10^4$ ct/min) was injected intraveously into 24 rats. Twenty-four hours later a tumour cell suspension ($5 \times 10^6$ cells) was injected into 12 animals; 12 control animals received the same volume of buffered saline. Five minutes after the tumour cell or saline injections the animals were killed by rapid intraveous injection of 0·5 ml of Nembutal (Abbott Laboratories Ltd.). Blood was obtained by heart puncture before death. The right lung, parts of the liver, the spleen and one kidney were removed. After determination of the wet weight the radioactivity was measured in a conventional scintillation counter and converted to ct/min/g tissue.

Twenty-four hours before the injection of $5 \times 10^6$ tumour cells, 40 animals were injected with labelled platelets as described. The animals were divided into 5 groups and pretreated with intravenous injections of either heparin (Weddel Pharmaceuticals Ltd.), dipyridamole (Persantin, Boehringer

TABLE I.—*Schedule of Treatment before the Intravenous Injection of* $5 \times 10^6$ *Tumour Cells*

| Treatment | Dose | Time before tumour injection (min) | No. of animals |
|---|---|---|---|
| Heparin | 750 u/kg b.w. | 15 | 10 |
| Dipyridamole | 30 mg/kg b.w. | 15 | 10 |
| Ancrod | 90 u/kg b.w. | 30 | 10 |
| Saline | 0·5 ml | 15 | 5 |
| Saline | 0·5 ml | 30 | 5 |

Ingelheim), ancrod (Arvin, Twyford Laboratories) or 0·9% saline as outlined in Table I. Five minutes after the tumour cell injection the animals were killed, and blood and lung radioactivity was determined according to the procedure mentioned above. Immediately before death a blood sample from the tail vein was obtained for platelet counts.

Five healthy control animals were injected with 750 u/kg body weight of heparin and platelet counts and whole blood clotting times were performed before and 15 min after the injection. Five additional control animals were injected with 90 u/kg body weight of ancrod and platelet counts were determined before and 30 min after the injection; the plasma fibrinogen level was estimated 30 min after the injection.

The significance of the results obtained in each experiment was established by the Student *t*-test and the probability (*P*) was estimated.

### RESULTS

The intramuscular transplantation of all tumour cell suspensions used in these experiments initiated solid tumour growth in healthy rats within 9–10 days, thus corresponding to the growth pattern of freshly obtained tumour cells. Triple frozen and thawed tumour cells failed to induce tumour growth when injected intramuscularly.

Fig. 1 demonstrates the mean number of blood platelets during the first 60 minutes following the intravenous injection of $7 \cdot 5 \times 10^6$ freshly prepared Walker carcinosarcoma 256 cells ($n = 10$) and cell fragments from the same amount of tumour cells ($n = 6$). The normal platelet count and its standard deviation were established from 25 untreated female rats (110–150 g). It is evident that there

FIG. 1.—Mean number of platelets following the intravenous injection of $7 \cdot 5 \times 10^6$ Walker carcinosarcoma 256 cells (○——○) and tumour cell fragments (□——□).

is no significant difference between the effect of freshly prepared tumour cells and tumour cell fragments upon the number of circulating platelets. The platelet count remains low during the period of observation. Twenty-four hours after the tumour cell injection the mean platelet count was 420,000 (±120,000) per mm³ and after 48 hours it had reached normal values again.

The mean number of platelets 15 minutes after the intravenous and intra-arterial injections of various amounts of tumour cells is demonstrated in Table II. Independent of the route of injection there is a close correlation between the number of tumour cells injected and the decrease in circulating platelets. However, the same amount of tumour cells which produced a marked thrombocytopenia if injected intravenously ($5 \times 10^6$ cells) did not affect the platelet count if

TABLE II.—*Platelet Counts in Rats 15 Minutes after Intravenous (i.v.) or Intra-arterial (i.a.) Injection of Various Amounts of Walker 256 Tumour Cells*

| Number of tumour cells injected | Route of injection | Platelets/mm³ | Standard deviation | No. of animals |
|---|---|---|---|---|
| $2 \cdot 5 \times 10^6$ | i.v. | 590000 | ±105000 | 5 |
| $5 \times 10^6$ | i.v. | 384000 | ±52000 | 8 |
| $7 \cdot 5 \times 10^6$ | i.v. | 198000 | ± 46000 | 10 |
| $1 \cdot 5 \times 10^7$ | i.a. | 685000 | ±122000 | 5 |
| $3 \times 10^7$ | i.a. | 176000 | ±95000 | 8 |
| $6 \times 10^7$ | i.a. | 16100 | ±6300 | 6 |
| Control | — | 831000 | ±114000 | 25 |

injected into the arterial side. A considerable increase in the tumour cell count is necessary to produce a comparable thrombocytopenia by intra-arterial injection (Table II).

The organ distribution of $^{51}Cr$ labelled platelets 5 minutes after the intravenous injection of $5 \times 10^6$ tumour cells is shown in Fig. 2. The decrease of blood activity and the increase of lung activity in the tumour cell injected animals ($n = 12$) is highly significant ($P$ in both cases $< 0 \cdot 01$) if compared with the control group ($n = 12$). The platelet distribution in the liver, spleen and kidneys is identical in the 2 groups.

Table III demonstrates the mean ct/min in the blood and the distribution of $^{51}Cr$ labelled platelets in the lungs of animals pretreated with various agents 5 minutes after the intravenous injection of $5 \times 10^6$ tumour cells. The radioactivity in the lungs and the blood radioactivity had an inverse relationship. Saline, dipyridamole and ancrod pretreatment did not influence the accumulation of platelets in the lungs nor their disappearance from the blood. These groups are statistically not different from each other. Heparin prevented the decrease of blood activity and the increase in lung activity if compared with the other treated groups ($P < 0 \cdot 01$). The blood activity in the various groups is closely related to the amount of circulating platelets, which is also demonstrated in Table III.

FIG. 2.—Distribution of $^{51}Cr$ labelled platelets in various organs 5 min after the intravenous injection of $5 \times 10^6$ Walker carcinosarcoma 256 cells or buffered saline.

The injection of 750 u/kg body weight of heparin into otherwise untreated animals ($n = 5$) prolonged the whole blood clotting time from 2·5 (± 0·7) minutes to over 30 minutes; the platelet count was not altered by the heparin treatment. Ninety u/kg body weight of ancrod injected intravenously revealed the absence of clottable fibrinogen 30 minutes after the injection ($n = 5$); the platelet count did not differ significantly from the initial values.

The plasma fibrinogen level was determined in 12 control and 12 tumour cell injected ($5 \times 10^6$ cells) animals. The tumour cell injected animals were bled 15 minutes ($n = 6$) and 30 minutes ($n = 6$) after the injection. The mean value for the control animals was 255 mg/100 ml (± 40 mg/100 ml) against a mean value in the tumour group of 230 mg/100 ml (± 45 mg/100 ml) at 15 minutes and 235 mg/100 ml (± 30 mg/100 ml) at 30 minutes. These dif-

TABLE III.—*Platelet Counts and Distribution of $^{51}Cr$-labelled Platelets in Blood and Lungs 5 Minutes after the Intravenous Injection of $5 \times 10^6$ Tumour Cells into Rats Pretreated with Various Agents*

| | ct/min × 10²/g tissue | | | | Platelets/mm³ | |
|---|---|---|---|---|---|---|
| | Lung | | Blood | | | |
| Treatment | Mean | s.d. | Mean | s.d. | Mean | s.d. |
| Heparin | 112 | 18 | 46 | 4·2 | 701000 | 112000 |
| Dipyridamole | 201 | 25 | 16 | 5·4 | 335000 | 51000 |
| Ancrod | 195 | 31 | 18 | 6·1 | 370000 | 54000 |
| Saline | 208 | 26 | 16 | 5·9 | 362000 | 49000 |

ferences are statistically insignificant. In all 12 plasma samples from the tumour animals the protamine precipitation test gave positive results, whereas in the control plasmas only one out of 12 was found positive.

The addition of a collagen suspension to rat platelet rich plasma and the recording of the changes in light transmission in the aggregation meter resulted in the well established curve for collagen induced platelet aggregation. The addition of tumour cell suspensions up to a concentration of $5 \times 10^7$ cells/ml did not induce platelet aggregation. Tumour cell fragments and extracts were equally ineffective in producing platelet aggregation. The examination of tumour cell platelet suspensions by phase contrast microscopy did not suggest any interaction between these 2 types of cells.

### DISCUSSION

Ultrastructural studies of embolic Walker carcinosarcoma 256 cells reaching the lung after intravenous injection revealed that the cells were arrested singly or in small groups in capillaries and arterioles. The presence of a fibrin-like meshwork around these cells was observed. However, electron microscopy studies failed to demonstrate significant amounts of polymerized fibrin but numbers of platelets were surrounding the tumour cells (Jones *et al.*, 1971). Since Gasic *et al.* (1968) reported that neuraminidase induced thrombocytopenia was associated with a significant reduction of experimental metastases in mice, some consideration has been directed towards a platelet–tumour cell interaction in the lodgement of haematogenous tumour cells (Gastpar, 1970; Kolenich, Mansour and Flynn, 1972; Gasic, Gasic and Murphy, 1972; Wood and Hilgard, 1972). None of the experimental data available allow any conclusion concerning the mechanism of platelet involvement in early metastasis formation and it seemed desirable to elucidate this phenomenon.

The immediate drop in the number of circulating blood platelets following the intravenous injection of a tumour cell suspension obviously represents the haematological counterpart to the morphological finding of tumour cells associated with platelet clusters (Jones *et al.*, 1971; Warren and Vales, 1972). The correlation of the platelet decrease and the number of tumour cells injected seems to confirm this assumption. Isotope studies demonstrated the rapid disappearance of the platelets from the circulation and their trapping in the lungs—the primary site of tumour cell lodgement. However, this phenomenon is not dependent upon the presence of living cells since the intravenous injection of non-viable tumour cell fragments initiated an identical decrease of circulating platelets.

In contrast to Warren's (1970) finding in the Chandler tube, neither fresh Walker carcinosarcoma 256 cells nor tumour cell fragments aggregated rat platelets *in vitro* in the aggregation meter. Furthermore, the injection of Walker carcinosarcoma 256 cells into the arterial side of the animals produced a thrombocytopenia only when the number of tumour cells was considerably increased. These two facts suggest that the tumour cell–platelet interaction is mediated *in vivo* by additional mechanisms which for their part are dependent upon local factors.

Pretreatment with dipyridamole in a dose which inhibits platelet aggregation (Cucuianu, Nichizawa and Mustard, 1971) was ineffective in preventing the tumour cell induced decrease of blood platelets. On the other hand, heparin completely inhibited the development of the thrombocytopenia. The lack of an increased platelet accumulation in the lungs of heparinized animals suggests that the formation of tumour cell associated platelet aggregates was inhibited by heparin, whereas dipyridamole was ineffective.

The presence of fibrin monomers indicated that, in spite of the unchanged plasma fibrinogen levels, intravascular fibrin formation accompanied the consumption of blood platelets. To study the behaviour of blood platelets in the absence of clottable fibrinogen, the animals were rendered afibrinogenaemic by the injection of ancrod (a fraction of the venom from the Malayan pit viper *Akistrodon rhodostoma*). Ancrod induces a state of hypofibrinogenaemia in various laboratory animals by formation of intravascular microclots which are rapidly removed from the circulation (Ashford, Ross and Southgate, 1968). The number and function of circulating platelets are not affected by this treatment (Davey and Lüscher, 1965). The identical pattern of the platelet decrease and their increased accumulation in the lung after tumour cell injection in afibrinogenaemic animals indicate that the formation of thrombin at the site of tumour cell lodgement is the crucial event. Thrombin induces intravascular platelet aggregation and the platelet release reaction earlier and at lower concentrations than it induces fibrin formation (Mustard and Packham, 1970). Ancrod prevents clot formation by removing the clottable protein; nevertheless, it does not interfere with the generation of thrombin. On the other hand, even low concentrations of thrombin initiate the formation of soluble intermediate polymers of the fibrinogen–fibrin conversion and this polymerizing fibrin becomes associated with platelets and causes their aggregation. Inhibitors of the platelet release reaction and of platelet aggregation, such as dipyridamole, do not prevent this interaction (Niewiarowsky *et al.*, 1972). Heparin of course, interferes with the formation of thrombin and therefore probably inhibited the development of the thrombocytopenia in this experimental model.

The degree of the thrombocytopenia in the present experiments was highly dependent upon the route of injection of the tumour cells, which indicates that local factors rather than tumour cell related factors are involved in the generation of thrombin. Ashford and Frieman (1968) demonstrated that minimal endothelial trauma results in platelet clusters associated with the surface of the endothelium. If the endogenous fibrinolytic response was inhibited by epsilon-amino caproic acid pretreatment these platelets were intimately associated with fibrin. In view of previous light microscopy studies (Wood, 1958) it seems justifiable to assume that tumour cells induce such minimal endothelial lesions at the site of their arrest in the blood vessel. Breaks in the plasma membrane, with exposure of cytoplasmic contents of the endothelial cells to the plasma, may lead to local activation of the clotting mechanism with formation of thrombin. Johnson *et al.* (1965) concluded from their ultrastructural studies that thrombin must form very early after endothelial damage and that it diffuses rapidly around the blood cells in that area. The fact that non-viable tumour cells and tumour cell membranes behave identically with viable tumour cells in regard to their effect upon circulating platelets further suggests that the interaction of the tumour cell membrane with the vascular endothelium and its subsequent damage is the primary event.

This work was supported by a grant from the " Deutsche Forschungsgemeinschaft ", Bad Godesberg, Germany.

### REFERENCES

AGOSTINO, D. & CLIFFTON, E. E. (1965) Trauma as a Cause of Localization of Blood Borne Metastases. Preventive Effect of Heparin and Fibrinolysis. *Ann. Surg.*, **161**, 97.

AGOSTINO, D. & CLIFFTON, E. E. (1968) The Growth and Transplantability of the Carcinosarcoma of Walker 256 in the Ascitic Form. *Experientia*, **24**, 166.

ASHFORD, A., ROSS, J. W. & SOUTHGATE, P. (1968) Pharmacology and Toxicology of a Defibrinating Substance from Malayan Pit Viper Venom. *Lancet*, i, 486.

ASHFORD, T. P. & FRIEDMAN, D. G. (1968) Platelet Aggregation at Sites of Minimal Endothelial Injury. An Electron Microscopic Study. *Am. J. Path.*, **53**, 599.

BRECHER, G. & CRONKITE, E. P. (1950) Morphology and Enumeration of Human Blood Platelets. *J. appl. Physiol.*, **3**, 365.

CLIFFTON, E. E. & AGOSTINO, D. (1965) The Effects of Fibrin Formation and Alterations in the Clotting Mechanism on the Development of Metastases. *Vasc. Dis.*, **2**, 43.

CLIFFTON, E. E., AGOSTINO, D., MADDEN, R. E. & BERECHID, J. N. (1971) Distribution in the Lungs of Labeled Walker 256 Carcinosarcoma Cells. An Autoradiographic Study with Tritiated Cytidine. *Archs Surg., Chicago*, **103**, 373.

CUCUIANU, M. P., NISHIZAWA, E. E. & MUSTARD, J. F. (1971) Effect of Pyrimido-pyrimidine Compounds on Platelet Function. *J. Lab. clin. Med.*, **77**, 958.

DACIE, J. V. & LEWIS, S. M. (1968) *Practical Haematology*. 4th Ed. London: Churchill.

DAVEY, M. G. & LÜSCHER, E. F. (1965) Actions of Some Coagulant Snake Venoms on Blood Platelets. *Nature, Lond.*, **207**, 730.

GASIC, G. J., GASIC, T. B. & STEWART, C. C. (1968) Antimetastatic Effects Associated with Platelet Reduction. *Proc. natn. Acad. Sci. U.S.A.*, **61**, 46.

GASIC, G. J., GASIC, T. B. & MURPHY, S. (1972) Antimetastatic Effect of Aspirin. *Lancet*, ii, 932.

GASTPAR, H. (1970) Stickiness of Platelets and Tumor Cells Influenced by Drugs. *Thromb. Diath. haemorrh.* Suppl., **42**, 291.

GRIFFITH, J. D. & SALSBURY, A. J. (1963) The Fate of Circulating Walker 256 Tumour Cells Injected Intravenously in Rats. *Br. J. Cancer*, **17**, 546.

JOHNSON, S. A., BALBOA, R. S., PEDERSON, H. J. & BUCKLEY, M. (1965) The Ultrastructure of Platelet Participation in Hemostasis. *Thromb. Diath. haemorrh.*, **13**, 65.

JONES, D. S., WALLACE, A. C. & FRASER, E. E. (1971) Sequence of Events in Experimental Metastases of Walker 256 Tumor: Light, Immunofluorescent and Electron Microscopic Observations. *J. natn. Cancer Inst.*, **46**, 493.

KOLENICH, J. J., MANSOUR, E. G. & FLYNN, A. (1972) Haematological Effects of Aspirin. *Lancet*, ii, 714.

MUSTARD, J. F. & PACKHAM, M. A. (1970) Factors Influencing Platelet Function: Adhesion, Release and Aggregation. *Pharmac. Rev.*, **22**, 97.

NIEWIAROWSKY, S., REGOECZI, E., STEWART, G. J., SENYI, A. F. & MUSTARD, J. F. (1972) Platelet Interaction with Polymerizing Fibrin. *J. clin. Invest.*, **51**, 685.

RATNOFF, O. D. & MENZIE, C. (1951) A New Method for the Determination of Fibrinogen in Small Samples of Plasma. *J. Lab. clin. Med.*, **3**, 316.

RYAN, J. J., KETCHAM, A. S. & WEXLER, H. (1968) Warfarin Treatment of Mice Bearing Autochthonous Tumors: Effect on Spontaneous Metastases. *Science, N.Y.*, **162**, 1493.

SANFELIPPO, M. J., STEVENS, D. J. & KOENIG, R. R. (1971) Protamine Sulfate Test for Fibrin Monomers. *Am. J. clin. Path.*, **56**, 166.

STEWART, H. L., SNELL, K. C., DUNHAM, L. J. & SCHLEYEN, S. M. (1959) Transplantable and Transmissable Tumors of Animals. *Atlas of Tumor Pathology*. Section XII, Fascicle 40. Washington: Armed Forces Institute of Pathology.

WARREN, B. A. (1970) The Ultrastructure of Platelet Pseudopodia and the Adhesion of Homologous Platelets to Tumour Cells. *Br. J. exp. Path.*, **51**, 570.

WARREN, B. A. & VALES, O. (1972) The Adhesion of Thromboplastic Tumour Emboli to Vessel Walls *in vivo*. *Br. J. exp. Path.*, **53**, 301.

WOOD, S. JR. (1958) Pathogenesis of Metastasis Formation Observed *in vivo* in the Rabbit Ear Chamber. *Archs Path.*, **66**, 550.

WOOD, S., JR, HOLYOKE, E. D. & YARDLEY, J. H. (1961) Mechanisms of Metastasis Production from Blood Borne Cancer Cells. *Can. Cancer Conf.*, **4**, 167.

WOOD, S., JR & HILGARD, P. (1972) Aspirin and Tumour Metastasis. *Lancet*, ii, 1416.


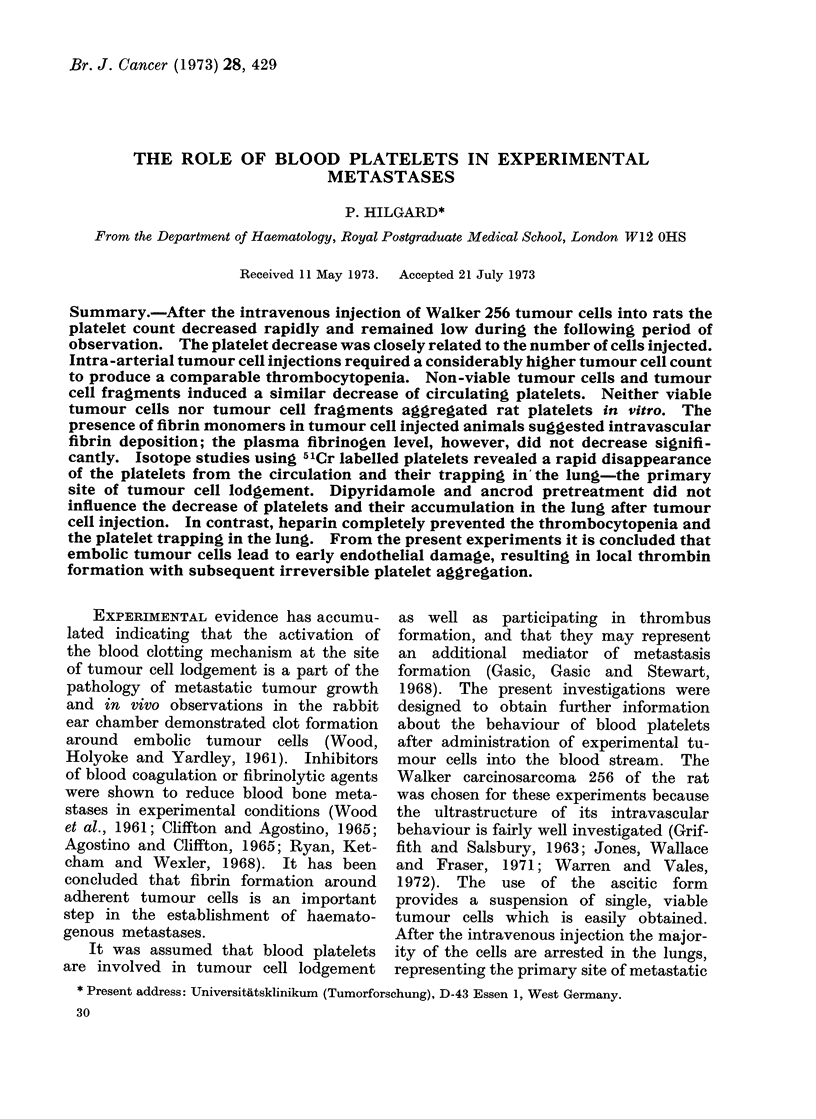

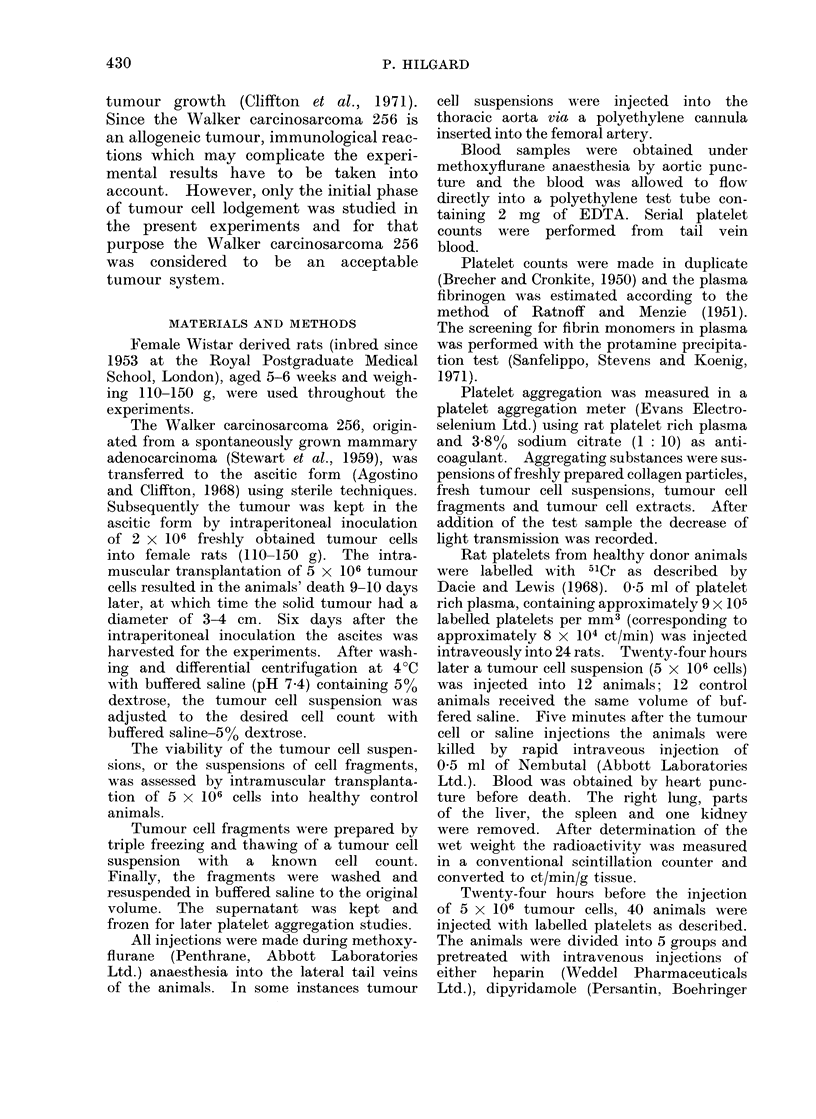

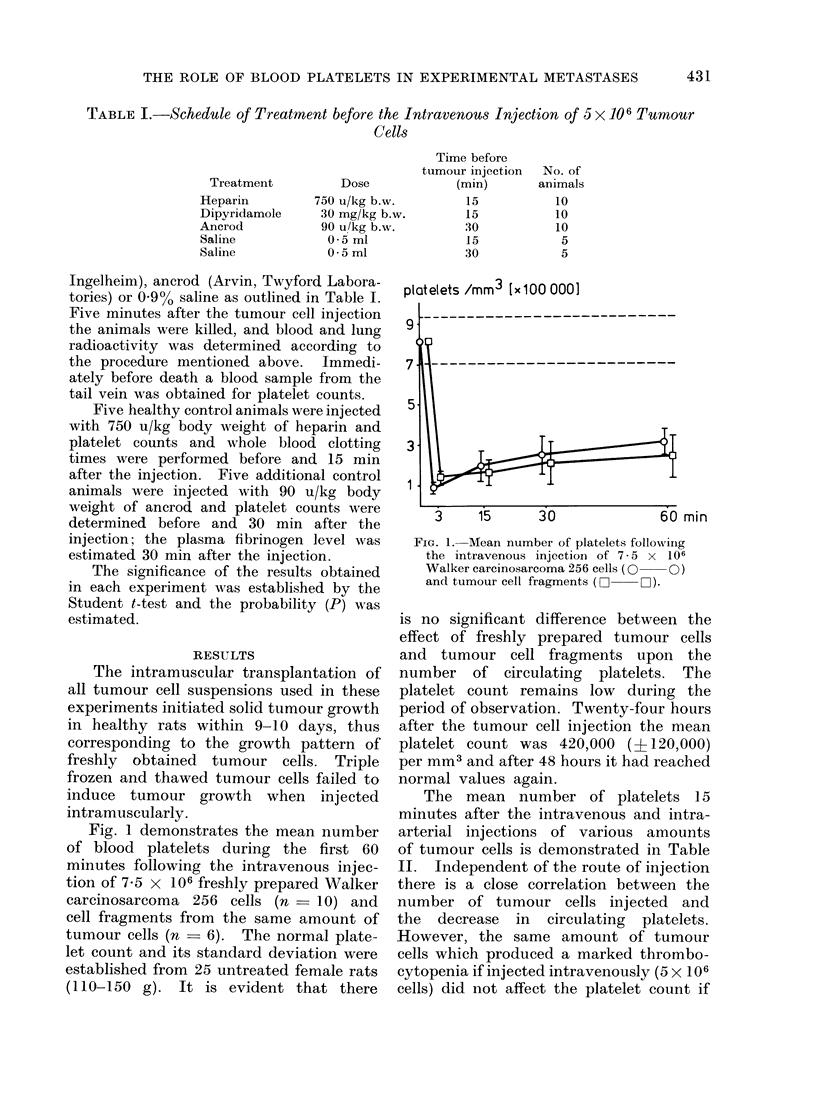

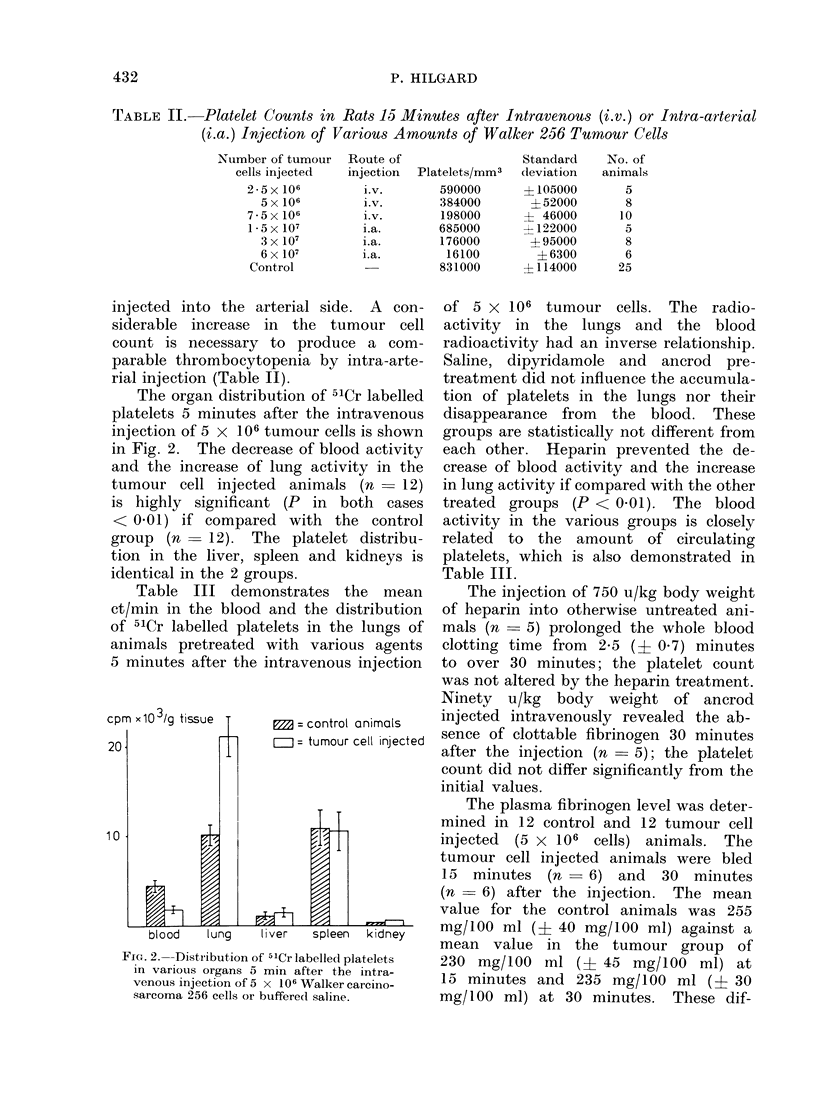

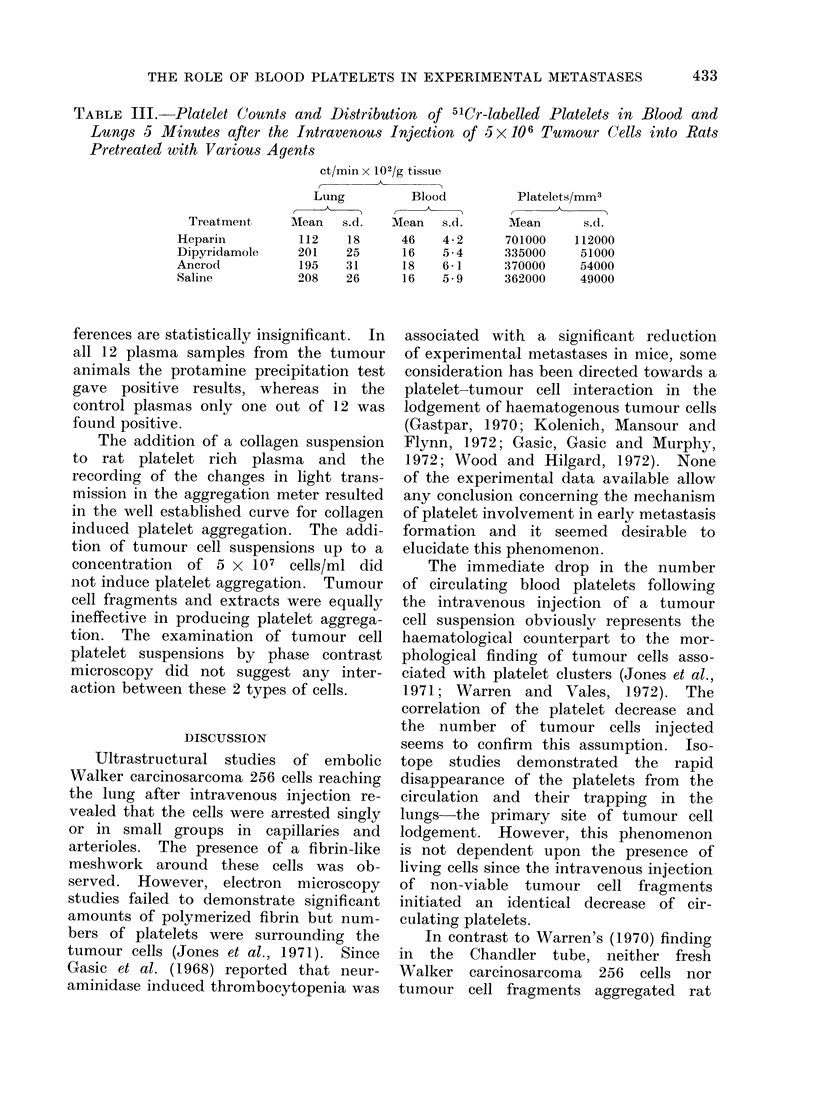

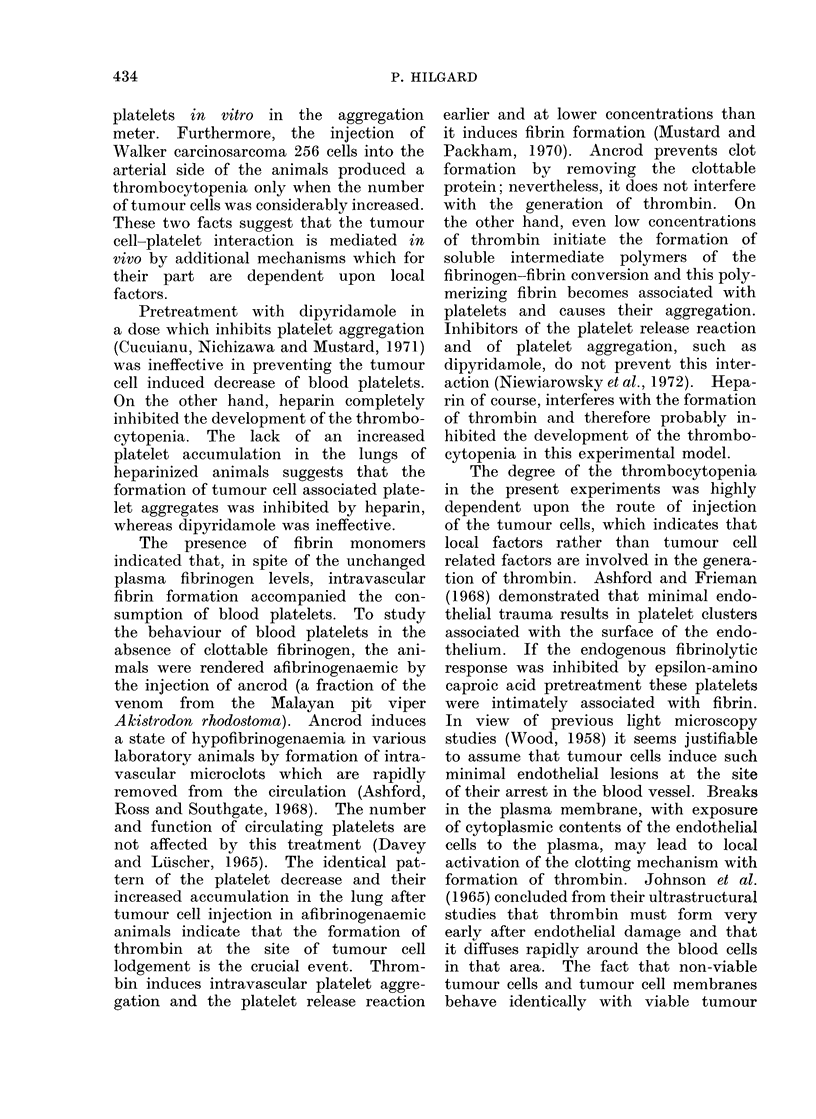

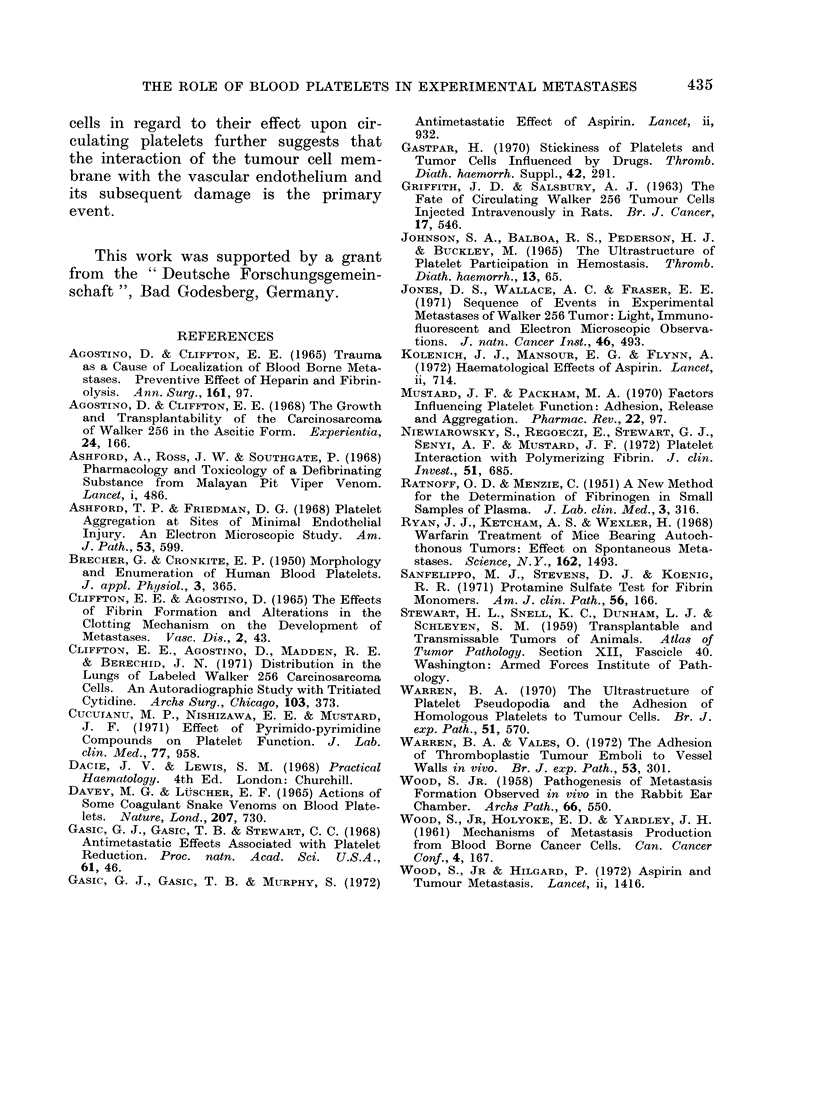


## References

[OCR_00744] AGOSTINO D., CLIFFTON E. E. (1965). TRAUMA AS A CAUSE OF LOCALIZATION OF BLOOD-BORNE METASTASES: PREVENTIVE EFFECT OF HEPARIN AND FIBRINOLYSIN.. Ann Surg.

[OCR_00750] Agostino D., Cliffton E. E. (1968). The growth and transplantability of the carcinosarcoma of Walker 256 in the ascitic form.. Experientia.

[OCR_00756] Ashford A., Ross J. W., Southgate P. (1968). Pharmacology and toxicology of a defibrinating substance from Malayan pit viper venom.. Lancet.

[OCR_00768] BRECHER G., CRONKITE E. P. (1950). Morphology and enumeration of human blood platelets.. J Appl Physiol.

[OCR_00773] CLIFFTON E. E., AGOSTINO D. (1965). THE EFFECTS OF FIBRIN FORMATION AND ALTERATIONS IN THE CLOTTING MECHANISM ON THE DEVELOPMENT OF METASTASES.. Vasc Dis.

[OCR_00779] Cliffton E. E., Agostino D., Madden R. E., Berechid J. N. (1971). Distribution in the lungs of labeled Walker 256 carcinosarcoma cells. An autoradiographic study with tritiated cytidine.. Arch Surg.

[OCR_00786] Cucuianu M. P., Nishizawa E. E., Mustard J. F. (1971). Effect of pyrimido-pyrimidine compounds on platelet function.. J Lab Clin Med.

[OCR_00796] Davey M. G., Lüscher E. F. (1965). Actions of some coagulant snake venoms on blood platelets.. Nature.

[OCR_00817] GRIFFITHS J. D., SALSBURY A. J. (1963). THE FATE OF CIRCULATING WALKER 256 TUMOUR CELLS INJECTED INTRAVENOUSLY IN RATS.. Br J Cancer.

[OCR_00807] Gasic G. J., Gasic T. B., Murphy S. (1972). Anti-metastatic effect of aspirin.. Lancet.

[OCR_00801] Gasic G. J., Gasic T. B., Stewart C. C. (1968). Antimetastatic effects associated with platelet reduction.. Proc Natl Acad Sci U S A.

[OCR_00823] JOHNSON S. A., BALBOA R. S., PEDERSON H. J., BUCKLEY M. (1965). THE ULTRASTRUCTURE OF PLATELET PARTICIPATION IN HEMOSTASIS.. Thromb Diath Haemorrh.

[OCR_00829] Jones D. S., Wallace A. C., Fraser E. E. (1971). Sequence of events in experimental metastases of Walker 256 tumor: light, immunofluorescent, and electron microscopic observations.. J Natl Cancer Inst.

[OCR_00836] Kolenich J. J., Mansour E. G., Flynn A. (1972). Haematological effects of aspirin.. Lancet.

[OCR_00841] Mustard J. F., Packham M. A. (1970). Factors influencing platelet function: adhesion, release, and aggregation.. Pharmacol Rev.

[OCR_00846] Niewiarowski S., Regoeczi E., Stewart G. J., Senyl A. F., Mustard J. F. (1972). Platelet interaction with polymerizing fibrin.. J Clin Invest.

[OCR_00852] RATNOFF O. D., MENZIE C. (1951). A new method for the determination of fibrinogen in small samples of plasma.. J Lab Clin Med.

[OCR_00857] Ryan J. J., Ketcham A. S., Wexler H. (1968). Warfarin treatment of mice bearing autochthonous tumors: effect on spontaneous metastases.. Science.

[OCR_00863] Sanfelippo M. J., Stevens D. J., Koenig R. R. (1971). Protamine sulfate test for fibrin monomers.. Am J Clin Pathol.

[OCR_00887] WOOD S. (1958). Pathogenesis of metastasis formation observed in vivo in the rabbit ear chamber.. AMA Arch Pathol.

[OCR_00876] Warren B. A. (1970). The ultrastructure of platelet pseudopodia and the adhesion of homologous platelets to tumour cells.. Br J Exp Pathol.

[OCR_00882] Warren B. A., Vales O. (1972). The adhesion of thromboplastic tumour emboli to vessel walls in vivo.. Br J Exp Pathol.

[OCR_00898] Wood S., Hilgard P. (1972). Aspirin and tumour metastasis.. Lancet.

